# Preoperative Prediction of Microvascular Invasion in Patients With Hepatocellular Carcinoma Based on Radiomics Nomogram Using Contrast-Enhanced Ultrasound

**DOI:** 10.3389/fonc.2021.709339

**Published:** 2021-09-07

**Authors:** Di Zhang, Qi Wei, Ge-Ge Wu, Xian-Ya Zhang, Wen-Wu Lu, Wen-Zhi Lv, Jin-Tang Liao, Xin-Wu Cui, Xue-Jun Ni, Christoph F. Dietrich

**Affiliations:** ^1^Department of Medical Ultrasound, Affiliated Hospital of Nantong University, Nantong, China; ^2^Department of Medical Ultrasound, Tongji Hospital, Tongji Medical College, Huazhong University of Science and Technology, Wuhan, China; ^3^Department of Artificial Intelligence, Julei Technology Company, Wuhan, China; ^4^Department of Diagnostic Ultrasound, Xiang Ya Hospital, Central South University, Changsha, China; ^5^Department of Internal Medicine, Hirslanden Clinic, Bern, Switzerland

**Keywords:** microvascular invasion, hepatocellular carcinoma, contrast-enhanced ultrasound, radiomics, nomogram

## Abstract

**Purpose:**

This study aimed to develop a radiomics nomogram based on contrast-enhanced ultrasound (CEUS) for preoperatively assessing microvascular invasion (MVI) in hepatocellular carcinoma (HCC) patients.

**Methods:**

A retrospective dataset of 313 HCC patients who underwent CEUS between September 20, 2016 and March 20, 2020 was enrolled in our study. The study population was randomly grouped as a primary dataset of 192 patients and a validation dataset of 121 patients. Radiomics features were extracted from the B-mode (BM), artery phase (AP), portal venous phase (PVP), and delay phase (DP) images of preoperatively acquired CEUS of each patient. After feature selection, the BM, AP, PVP, and DP radiomics scores (Rad-score) were constructed from the primary dataset. The four radiomics scores and clinical factors were used for multivariate logistic regression analysis, and a radiomics nomogram was then developed. We also built a preoperative clinical prediction model for comparison. The performance of the radiomics nomogram was evaluated *via* calibration, discrimination, and clinical usefulness.

**Results:**

Multivariate analysis indicated that the PVP and DP Rad-score, tumor size, and AFP (alpha-fetoprotein) level were independent risk predictors associated with MVI. The radiomics nomogram incorporating these four predictors revealed a superior discrimination to the clinical model (based on tumor size and AFP level) in the primary dataset (AUC: 0.849 *vs*. 0.690; p < 0.001) and validation dataset (AUC: 0.788 *vs*. 0.661; p = 0.008), with a good calibration. Decision curve analysis also confirmed that the radiomics nomogram was clinically useful. Furthermore, the significant improvement of net reclassification index (NRI) and integrated discriminatory improvement (IDI) implied that the PVP and DP radiomics signatures may be very useful biomarkers for MVI prediction in HCC.

**Conclusion:**

The CEUS-based radiomics nomogram showed a favorable predictive value for the preoperative identification of MVI in HCC patients and could guide a more appropriate surgical planning.

## Introduction 

Hepatocellular carcinoma (HCC) is the most common primary hepatic malignancy and ranks third among all cancer-related deaths (1, 2). It has always been a major international health problem. Hepatectomy is recognized as the preferred treatment for primary HCC (3). However, recurrence occurs in 30%–50% of patients within 2 years after surgery, resulting in a lower overall survival rate (4). Therefore, it is very important to detect high-risk factors for early recurrence before surgery to enable the formulation of individualized treatment plans.

The definition of microvascular invasion (MVI) is the presence of microscopic metastatic hepatocellular carcinoma emboli within the smaller intrahepatic vessels (5). Some studies have confirmed that MVI is an essential determinant for predicting early recurrence and evaluating the long-term survival of HCC patients (6, 7). The presence of MVI is considered an aggressive pathological indicator (8). Larger resection margins are required for hepatectomy in high-risk patients with MVI (9). Accurate assessment of the presence of MVI before surgery can help surgeons choose appropriate surgical methods. Unfortunately, unlike macrovascular invasion, which can usually be detectable with preoperative imaging, MVI can only be determined according to postoperative pathological specimens (10). Preoperative biopsy is also unreliable due to sampling errors (11).

Imaging examination is an indispensable means of the preoperative evaluation of HCC, some studies have attempted to assess the relationship between preoperative imaging features and MVI status. Several recent reports have suggested that tumor size/number, non-smooth tumor margins, arterial peritumoral enhancement, higher mean kurtosis values, irregular circular enhancement, and radiological characteristics of the capsule may serve as predictors of MVI (12–15). Although these imaging features represent different rates of evaluation, the identification of imaging features mainly depends on the subjective judgment of the radiologist. The accuracy of diagnosis will be affected by the differences in the experience of radiologists. Therefore, a quantitative method is needed to identify MVI non-invasively and accurately before operation.

Radiomics is a process of converting images containing pathophysiology-related information into mineable high-dimensional data, enabling the quantification of diseases using unique imaging algorithms for the diagnosis, prediction, and prognostic evaluation at the molecular level (16–18). Previous studies have demonstrated the potential of radiomics to pre-operatively predict the status of MVI in patients with HCC (19–22). However, most of the radiomics signatures in these studies were based on computed tomography (CT) or magnetic resonance imaging (MRI). Compared with contrast-enhanced CT/MRI, contrast-enhanced ultrasound (CEUS) is a real-time imaging technology with no radiation and fewer limitations in liver examination (23–25). Zhou et al. reported that combined with the tumor number and tumor size, the washout rate of CEUS was significantly associated with the MVI status of HCC patients (26). To better interpret CEUS, we built a radiomics strategy.

Nomograms can be used for the multi-index joint diagnosis or prediction of disease onset or progression. Some studies have demonstrated that the nomograms incorporating clinical risk predictors such as serum α-fetoprotein level (AFP), tumor size, and platelet count (PLT) can be helpful in predicting preoperative MVI status for HCC (13, 27–29). To the best of our knowledge, there have been no previous studies to determine whether a nomogram containing CEUS radiomics would allow a superior prediction of the MVI status.

Thus, the aim of the present study was to develop and validate a radiomics nomogram that is based on the CEUS imaging and clinical risk factors for a preoperative prediction of the MVI status in patients with HCC.

## Materials and Methods

### Patients

This retrospective study was approved by the Institutional Review Board who waived the requirement of informed consent. For the datasets, we assessed the Xiangya Hospital Central South University medical records database between September 2016 and March 2020 to identify patients with a histologically confirmed HCC who underwent surgical resection. The inclusion criteria were as follows: (1) pathologically confirmed primary HCC after hepatic resection; (2) MVI status was confirmed by hepatectomy and histopathological results; (3) CEUS examinations were performed within the two weeks before surgery; (4) solitary tumor; and (5) no previous liver surgery or other treatments had been performed for the suspected HCC lesion. The exclusion criteria included: (1) preoperative anticancer therapy (e.g., radiotherapy, radiofrequency ablation, or transcatheter arterial chemoembolization) before CEUS examination; (2) recurrent HCC; (3) the CEUS image quality of target tumor was unsatisfactory for evaluation; and (4) incomplete clinico-pathological data. The flow diagram of the study population is presented in **Supplementary Figure A1**.

Patients who met the inclusion criteria were randomly allocated to a primary dataset and a validation dataset. The primary and validation datasets comprised 192 patients (166 men and 26 women; mean age, 55.1 ± 11.1 years; range, 27–83 years) and 121 patients (99 men and 22 women; mean age, 55.37 ± 12.1 years; range, 21–83 years), respectively.

### Clinical and Pathologic Data

Baseline clinical information, including sex, age, tumor size, hepatitis, cholelithiasis, serum liver function as well as tumor markers, were derived from the medical records. Serological data including alanine aminotransferase (ALT), AFP, aspartate aminotransferase (AST), PLT, international normalized ratio (INR), albumin (ALB), total bilirubin (TBIL), and direct bilirubin (DBIL) were obtained a week before the surgery. We also collected postoperative pathological information, including the presence of MVI, pathologic differentiation of HCC (well, moderate, or poor according to the WHO histologic grade system), and the presence of liver cirrhosis. Positive MVI refers to cancer cell nests within the vascular lumen that can only be observed under the microscopy.

### Contrast-Enhanced Ultrasound Examination

Image acquisition was performed within 2 weeks preoperatively. The CEUS images were acquired with the Aloka ARIETTA 70 (Aloka, Japan, C251 abdominal probe) ultrasound diagnostic instrument. All the CEUS examinations were performed by one of three experienced radiologists (each of whom had at least 15 years of hepatic CEUS experience).

First, the target tumor was detected and assessed by B-mode (BM) ultrasound, the transducer was fixed when the image showed the largest cross-section of the tumor, the maximum diameter measured was taken as the size of the tumor. Then, 2.4 mL of the second-generation ultrasound contrast agent (SonoVue, Bracco, Milan, Italy) was injected intravenously *via* the antecubital vein, followed by flushing with 5 mL of 0.9% normal saline solution. The timer was started immediately while the contrast agent was being injected. The target lesion was continuously observed on the largest cross-section, and each patient saved at least 4 minutes of digital movie clips on the hard disk. All the digital cine clips were recorded as digital imaging data and communications in medicine (DICOM) format and stored into the Picture Archiving and Communication Systems (PACS). Arterial phase (AP) images, portal venous phase (PVP) images, and delay phase (DP) images were obtained at 0–30 s, 31–120 s, and 121–240 s after intravenous injection of the contrast, respectively.

### Tumor Segmentation and Radiomics Feature Extraction

Two board-certified radiologists (radiologist 1 and radiologist 2), both with more than 10 years of experience in abdominal CEUS interpretation and blinded to the pathological results and clinical data, independently reviewed the CEUS documents, including all the digital movie clips from this study. For each patient, four images were selected for analysis, including one of BM (before the start of CEUS), one of AP (approximately 25 s after contrast injection), one of PVP (approximately 60 s after contrast injection), and one of DP (approximately 180 s after contrast injection). All four images showed the largest cross-section of the tumor. The slice chosen for delineating the lesion was confirmed by the two radiologists in consensus. Regions of interest (ROI) were annotated manually around the target lesion margin on the selected BM, AP, PVP, and DP images using an open-source software (ITK-SNAP 3.8.0; http://www.itksnap.org) by radiologist 1. The histogram, morphology, intensity, laws, wavelet, and texture features were extracted by using an open-source software (Pyradiomics; http://pyradiomics.readthedocs.io/en/latest/index.html) through computing algorithms and stored as comma separated values (CSVs).

To evaluate the inter-observer and intra-observer reproducibility, 50 patients and their corresponding BM, AP, PVP, and DP images were randomly selected and independently delineated by the two radiologists (twice by radiologist 1 with an interval of 2 weeks and once by radiologist 2). After features extraction, the intraclass and interclass correlation coefficients (ICCs) were applied to assess the inter-observer and intra-observer reproducibility of the extracted features from the two radiologists. Features with an ICC < 0.80 were eliminated in the subsequent analyses.

### Microvascular Invasion Status-Related Feature Selection and Radiomics Score Building

The Spearman rank-order correlation coefficient was implemented to evaluate the correlation and redundancy of radiomics features. The redundant features were eliminated with a Spearman rank-order correlation coefficient ≥ 0.8. Thereafter, the remaining features were selected by applying the minimum redundancy maximum relevance (mRMR) algorithm. Then, the key features related to the MVI status were selected by the least absolute shrinkage and selection operator (LASSO) logistic regression method using a five-fold cross validation after mRMR algorithm in the primary dataset. The LASSO algorithm was applied to weigh the linear combination of the selected features to generate a radiomics score (Rad-score). The formula for the BM, AP, PVP, and DP radiomics scores were established using the respective selected features. Then, the Mann-Whitney U test was applied in the primary and validation datasets to evaluate the potential association between the Rad-scores and MVI status.

### Ultrasound Radiomics Nomogram Construction and Validation

To identify the clinical risk factor associated with the MVI status, we performed univariate analyses of the clinical parameters. Chi-square test was used on categorical variables and Student’s t independent test was used on continuous variables. We further implemented a multivariable logistic regression analysis of the Rad-scores and independent clinical risk factors, variable selection was implemented with p-values below 0.05 as the preservation criteria to confirm the ultimate predictors for the MVI status. Then, a radiomics nomogram was constructed based on the multivariable logistic regression analysis in the primary dataset. For comparison, we developed a clinical prediction model that only incorporated the independent clinical risk factors.

The calibration curve and Hosmer-Lemeshow test were performed to evaluate the calibration of the radiomics nomogram. The discrimination performance and the clinical usefulness of the nomogram were evaluated using receiver operating characteristic (ROC) curve analysis and Decision curve analysis (DCA), respectively. The difference between areas under the curve (AUCs) was compared by the DeLong test. For clinical use, the total score of each patient (defined as Nomo-score) was calculated according to the radiomics nomogram scoring method. Thereafter, the optimal cut-off value was assessed by maximizing the Youden index. The prediction performance of the optimal cut-off value of the total score was evaluated *via* the ROC, accuracy, sensitivity, specificity, positive and negative likelihood ratios as well as predictive values.

### Statistical Analysis

All statistical analysis was conducted with the R software 3.6.1 (RStudio Inc.) and SPSS 24.0 software (SPSS Inc., Chicago, IL). Categorical variables were expressed as numbers or percentages, and continuous variables were expressed as mean ± SD or medians. The baseline clinical and pathologic data differences were compared by chi-square test for categorical variables and the Student’s t test or Mann-Whitney U test for continuous variables as appropriate between the primary dataset and validation dataset. All two-sided p-values less than 0.05 were considered statistically significant. The packages of R3.6.1 that were used are presented in **Supplementary Table A1**.

## Results

### Clinico-Pathological Information

**Figure 1:**
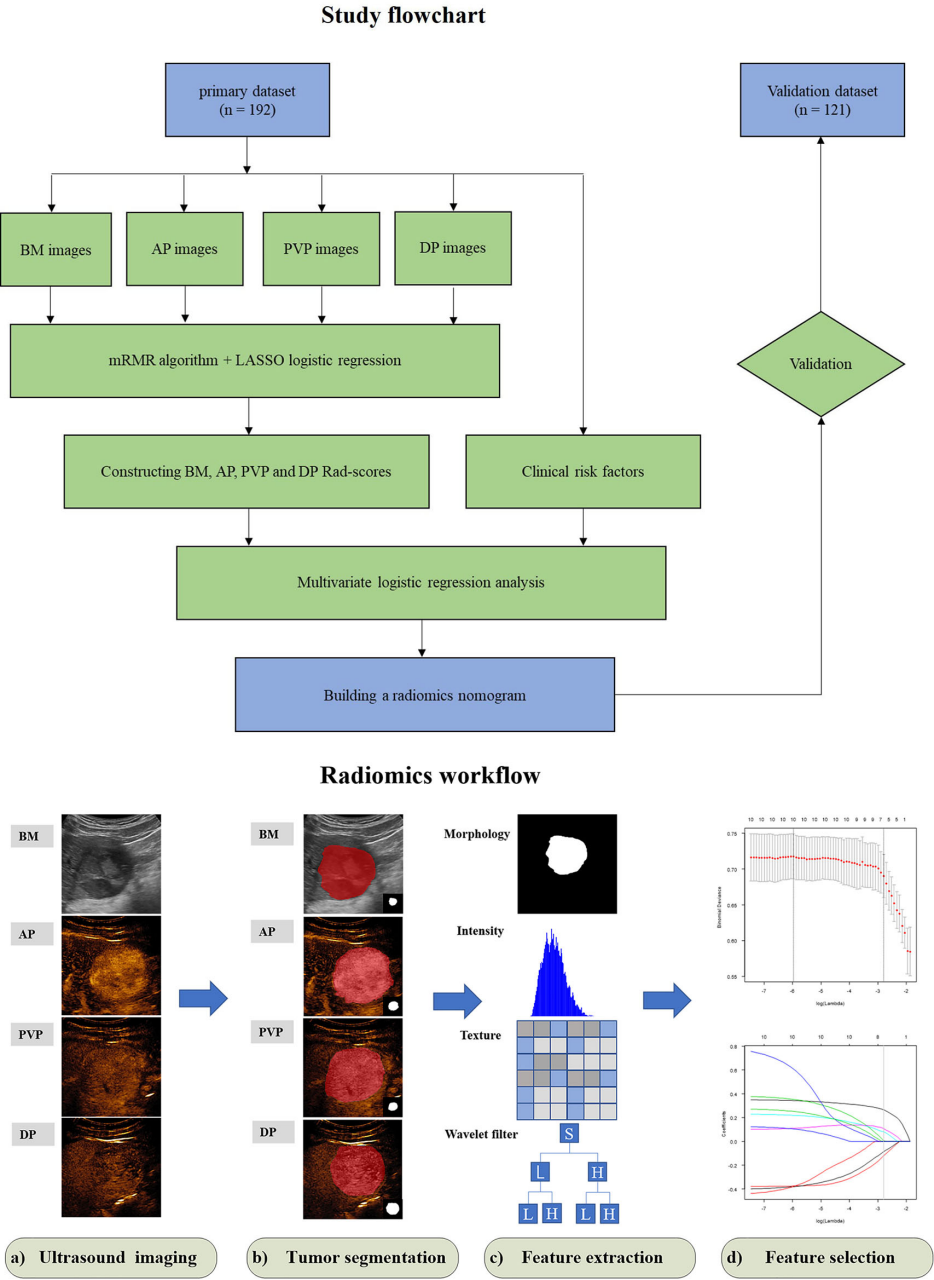
Study flowchart of radiomics nomogram modeling for the MVI status prediction in patients with HCC and radiomics workflow. BM, B-mode; AP, arterial phase; PVP, portal venous phase; DP, delay phase; Rad-score, radiomics score; mRMR, minimum redundancy maximum relevance; LASSO, least absolute shrinkage and selection operator.

The study flow chart is presented in **Figure 1**. The detailed clinico-pathological information of the two datasets is summarized in **Tables 1**, **2**. Positive MVI patients accounted for 41.1% (79/192) and 40.5% (49/121) of the primary and validation datasets, respectively. There was no significant difference between the two datasets in the presence of MVI (p = 0.909) or other clinicopathological characteristics. Univariate analysis revealed that the tumor size and AFP level were significantly different between the MVI positive and MVI negative groups in the primary dataset (**Table 2**). Thus, we constructed a clinical model for predicting the MVI status using multivariate logistic regression analysis based on the two clinical risk predictors.

**Table 1:** Clinicopathological characteristics in the primary and the validation datasets.

Characteristic	Primary dataset (n = 192)	Validation dataset (n = 121)	P-value
Gender			0.267
Male	166 (86.5)	99 (81.8)	
Female	26 (13.5)	22 (18.2)	
Age, mean ± SD, years	55.1 ± 11.0	55.37 ± 12.1	0.840
Tumor size			0.103
<5 cm	121 (63.0)	65 (53.7)	
≥5 cm	71 (37.0)	56 (46.3)	
MVI status			0.909
Positive	79 (41.1)	49 (40.5)	
Negative	113 (58.9)	72 (59.5)	
Pathologic grade			0.839
Well	30 (15.6)	22 (18.2)	
Moderately	123 (64.1)	75 (62.0)	
Poorly	39 (20.3)	24 (19.8)	
Cirrhosis			0.568
Positive	122 (63.5)	73 (60.3)	
Negative	70 (36.5)	48 (39.7)	
Cholelithiasis			0.993
Positive	38 (19.8)	24 (19.8)	
Negative	154 (80.2)	97 (80.2)	
Hepatitis			0.272
Positive	153 (79.7)	90 (74.4)	
Negative	39 (20.3)	31 (25.6)	
ALT (U/L)			0.808
≤40	109 (56.8)	67 (55.4)	
>40	83 (43.2)	54 (44.6)	
AST (U/L)			0.740
≤35	83 (43.2)	50 (41.3)	
>35	109 (56.8)	71 (58.7)	
AFP (μg/L)			0.203
≤20	74 (38.5)	58 (47.9)	
20–400	57 (29.7)	34 (28.1)	
≥400	61 (31.8)	29 (24.0)	
PLT (10^9^/L)			0.674
<100	56 (29.2)	38 (31.4)	
≥100	136 (70.8)	83 (68.6)	
ALB (g/L)			0.469
<40	84 (43.75)	58 (47.9)	
≥40	108 (56.25)	63 (52.1)	
INR			0.748
≤1.2	149 (77.6)	92 (76.0)	
>1.2	43 (22.4)	29 (24.0)	
TBIL (μmol/L)			0.491
≤17.1	131 (68.2)	78 (64.5)	
>17.1	61 (31.8)	43 (35.5)	
DBIL (μmol/L)			0.640
≤6.8	102 (53.1)	61 (50.4)	
>6.8	90 (46.9)	60 (49.6)	
BM rad-score, median (interquartile range)	-4.06 (-7.04 to -1.12)	-3.69 (-6.43 to -1.44)	0.648
AP rad-score, median (interquartile range)	-3.54 (-4.30 to -2.89)	-3.48 (-4.00 to -2.58)	0.256
PVP rad-score, median (interquartile range)	-3.98 (-9.23 to 1.08)	-2.17 (-7.09 to 2.27)	0.115
DP rad-score, median (interquartile range)	-3.50 (-3.97 to -3.03)	-3.42 (-3.97 to -2.96)	0.336

ALT, alanine aminotransferase; AST, aspartate aminotransferase; AFP, α-fetoprotein; PLT, platelets count; ALB, albumin; INR, international normalized ratio; TBIL, total bilirubin; DBIL, direct bilirubin; BM, B-mode; AP, arterial phase; PVP, portal venous phase; DP, delay phase; Rad-score, radiomics score. Unless otherwise specified, data in parentheses are percentages.

**Table 2:** Preoperative predictors for MVI in the primary and the validation datasets.

Characteristic	Primary dataset No. (%)			Validation dataset No. (%)		
	MVI (+)	MVI (-)	P value	MVI (+)	MVI (-)	P-value
Gender			0.467			0.600
male	70 (88.6)	96 (85.0)		39 (79.6)	60 (83.3)	
female	9 (11.4)	17 (15.0)		10 (20.4)	12 (16.7)	
Age, mean ± SD, years	54.0 ± 11.5	55.9 ± 10.6	0.224	53.8 ± 13.7	56.5 ± 10.8	0.232
Tumor size			<0.001			0.018
<5 cm	37 (46.8)	84 (74.3)		20 (40.8)	45 (62.5)	
≥5 cm	42 (53.2)	29 (25.7)		29 (59.2)	27 (37.5)	
Cirrhosis			0.503			0.093
Positive	48 (60.8)	74 (65.5)		34 (69.4)	39 (54.2)	
Negative	31 (39.2)	39 (34.5)		15 (30.6)	33 (45.8)	
Cholelithiasis			0.893			0.552
Positive	16 (20.3)	22 (19.5)		11 (22.4)	13 (18.1)	
Negative	63 (79.7)	91 (80.5)		38 (77.6)	59 (81.9)	
Hepatitis			0.282			0.132
Positive	60 (82.3)	93 (86.7)		40 (81.6)	50 (69.4)	
Negative	19 (17.7)	20 (13.3)		9 (18.4)	22 (30.6)	
ALT (U/L)			0.584			0.961
≤40	43 (54.4)	66 (58.4)		27 (55.1)	40 (55.6)	
>40	36 (45.6)	47 (41.6)		22 (44.9)	32 (44.4)	
AST (U/L)			0.733			0.222
≤35	33 (41.8)	50 (44.2)		17 (34.7)	33 (45.8)	
>35	46 (58.2)	63 (55.8)		32 (65.3)	39 (54.2)	
AFP (μg/L)			0.017			0.006
≤20	24 (30.4)	50 (44.2)		20 (40.8)	38 (52.8)	
20–400	21 (26.6)	36 (31.9)		10 (20.4)	24 (33.3)	
≥400	34 (43.0)	27 (23.9)		19 (38.8)	10 (13.9)	
PLT (10^9^/L)			0.192			0.580
<100	19 (24.1)	37 (32.7)		14 (28.6)	24 (33.3)	
≥100	60 (75.9)	76 (67.3)		35 (71.4)	48 (66.7)	
ALB (g/L)			0.897			0.575
<40	35 (44.3)	49 (43.4)		25 (51.0)	33 (45.8)	
≥40	44 (55.7)	64 (56.6)		24 (49.0)	39 (54.2)	
INR			0.914			0.328
≤1.2	61 (77.2)	88 (77.9)		35 (71.4)	57 (79.2)	
>1.2	18 (22.8)	25 (22.1)		14 (28.6)	15 (20.8)	
TBIL (μmol/L)			0.108			0.001
≤17.1	59 (74.7)	72 (63.7)		23 (46.9)	55 (76.4)	
>17.1	20 (25.3)	41 (36.3)		26 (53.1)	17 (23.6)	
DBIL (μmol/L)			0.139			0.035
≤6.8	47 (59.5)	55 (48.7)		19 (38.8)	42 (58.3)	
>6.8	32 (40.5)	58 (51.3)		30 (61.2)	30 (41.7)	
BM rad-score, median (interquartile range)	-2.10 (-4.68 to 1.23)	-5.45 (-8.22 to -2.61)	<0.001	-2.24 (-4.80 to 2.01)	-4.72 (-7.20 to -2.25)	<0.001
AP rad-score, median (interquartile range)	-3.07 (-3.88 to -2.04)	-3.96 (-4.65 to -3.32)	<0.001	-2.85 (-3.64 to -2.15)	-3.68 (-4.39 to -3.20)	<0.001
PVP rad-score, median (interquartile range)	0.32 (-3.92 to 6.36)	-7.04 (-12.50 to -3.18)	<0.001	1.83 (-2.79 to 4.47)	-5.44 (-9.63 to -0.52)	<0.001
DP rad-score, median (interquartile range)	-3.13 (-3.52 to -2.82)	-3.75 (-4.22 to -3.28)	<0.001	-3.09 (-3.62 to -2.89)	-3.58 (-4.05 to -3.16)	0.001

ALT, alanine aminotransferase; AST, aspartate aminotransferase; AFP, α-fetoprotein; PLT, platelets count; ALB, albumin; INR, international normalized ratio; TBIL, total bilirubin; DBIL, direct bilirubin. BM, B-mode; AP, arterial phase; PVP, portal venous phase; DP, delay phase; Rad-score, radiomics score; Unless otherwise specified, data in parentheses are percentages.

### Establishment of Ultrasound Radiomics Score

A set of 479 radiomics features were extracted from the BM, AP, PVP, and DP images of each patient. Favorable inter-observer and intra-observer reproducibility of feature extraction were achieved, with 90.2% (432) of the BM features, 89.4% (428) of the AP features, 93.1% (446) of the PVP features, and 82.5% (395) of the DP features had an intra-observer ICCs ≥ 0.80, and 90.2% (432) of the BM features, 93.5% (448) of the AP features, 92.5% (443) of the PVP features, and 95.4% (457) of the DP features had an inter-observer ICCs ≥ 0.80. For BM, six features were selected after mRMR algorithm and LASSO regression in the primary dataset for radiomics score construction (**Supplementary Figures A2A, B**). Similarly, two, eight, and nine radiomics features were finally selected as the potential predictors by mRMR algorithm and LASSO regression for the AP, PVP, and DP radiomics score construction, respectively (**Supplementary Figures A2C, D–H**). The calculation formulas of the BM, AP, PVP, and DP radiomics scores are provided in **Supplementary A1**. The BM, AP, PVP, and DP Rad-scores were all significantly higher in the MVI positive group in both the primary and validation datasets than those in the MVI negative group (**Table 2**). The performance of the four Rad-scores in distinguishing MVI-positive and MVI-negative patients are provided in **Supplementary Table 1**.

### Modeling and Evaluation of the Radiomics Nomogram

**Figure 2:**
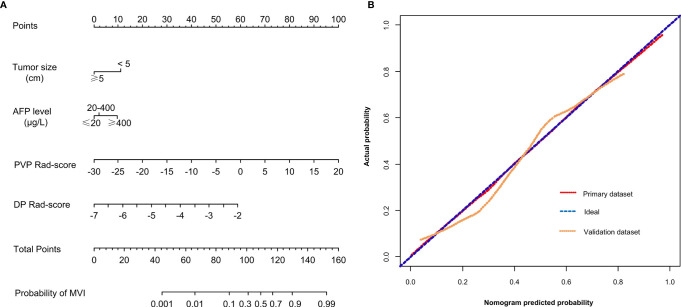
**(A)** A radiomics nomogram combining the AFP level, tumor size, PVP Rad-score, and DP Rad-score. **(B)** Calibration curves of the radiomics nomogram in the primary and validation datasets. AFP, α-fetoprotein; PVP, portal venous phase; DP, delay phase; Rad-score, radiomics score.

The PVP Rad-score, DP Rad-score, AFP level, and tumor size were identified as independent risk predictors of the MVI status in HCC patients by the results of the multivariate logistic regression analysis (**Table 3**). Thus, we constructed a radiomics nomogram incorporating the above four independent risk predictors (**Figure 2A**). The Hosmer-Lemeshow test (P = 0.872 and 0.606 for the primary and validation datasets, respectively) and calibration curve revealed a good calibration of the radiomics nomogram for predicting the MVI status in the primary and validation datasets (**Figure 2B**).

**Table 3:** Variables and coefficients of the radiomics nomogram and clinical model.

Intercept and variable	Clinical model			Radiomics nomogram		
	β	Odds ratio (95% CI)	P-value	β	Odds ratio (95% CI)	P-value
Intercept	-1.263			4.744		
Tumor size (≥5 cm)	1.204	3.334 (1.775 to 6.263)	<0.001	-1.170	0.310 (0.116 to 0.833)	0.020
AFP (μg/L)						
≤20		Reference			Reference	
20–400	0.358	1.430 (0.669 to 3.062)	0.357	0.243	1.276 (0.523 to 3.111)	0.593
≥400	1.006	2.734 (1.313 to 5.695)	0.007	1.029	2.797 (1.164 to 6.726)	0.022
BM Rad-score	NA	NA	NA	NA	NA	NA
AP Rad-score	NA	NA	NA	NA	NA	NA
PVP Rad-score	NA	NA		0.214	1.239 (1.138 to 1.347)	<0.001
DP Rad-score	NA	NA		1.261	3.529 (1.687 to 7.382)	0.001
AUC						*P value
Primary dataset	0.690 (0.615 to 0.766)			0.849 (0.795 to 0.902)		<0.001
Validation dataset	0.661 (0.561 to 0.760)			0.788 (0.704 to 0.872)		0.008

*P value represents the difference of AUC between the radiomics nomogram and clinical model. AFP, α-fetoprotein; BM, B-mode; AP, arterial phase; PVP, portal venous phase; DP, delay phase; Rad-score, radiomics score; AUC, area under the receiver operating characteristic curve; NA, not available.

**Figure 3:**
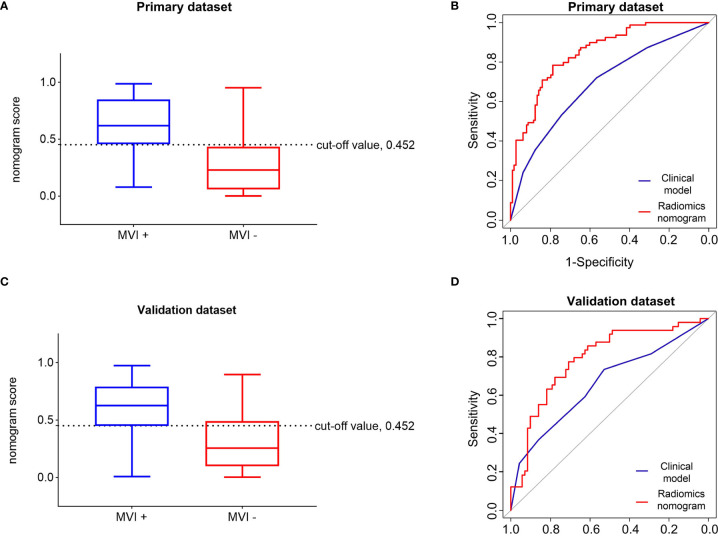
Performance of the radiomics nomogram in predicting the MVI status of patients with hepatocellular carcinoma. Differential diagnosis of MVI+ and MVI− groups with the cut-off value of Nom-score of 0.452 in the primary **(A)** and validation **(C)** datasets. Receiver operating characteristic curves of the radiomics nomogram and clinical model in the primary **(B)** and validation **(D)** datasets. MVI+, patients with microvascular invasion; MVI−, patients without microvascular invasion.

The optimal threshold of the Nomo-score to identify the MVI status was identified to be 0.452 according to the Youden index, and the performance of using the radiomics nomogram to predict the MVI status with the recommended cut-off value are summarized in **Table 4**. An AUC of 0.849 (95% CI, 0.795–0.902) for the primary dataset and 0.788 (95% CI, 0.704–0.872) for the validation dataset demonstrated a good discrimination ability of the nomogram (**Figure 3**).

**Table 4:** Performance of the radiomics nomogram and clinical model for evaluating the preoperative MVI status.

Variable	Value (95% CI)			
	Clinical model		Radiomics nomogram	
	Primary dataset	Validation dataset	Primary dataset	Validation dataset
Cut-off value	0.362	0.362	0.452	0.452
AUC	0.690 (0.615 to 0.766)	0.661 (0.561 to 0.760)	0.849 (0.795 to 0.902)	0.788 (0.704 to 0.872)
Sensitivity, %	72.15 (61.99 to 82.28)	73.47 (61.22 to 85.71)	78.48 (69.62 to 87.34)	75.51 (63.27 to 87.76)
Specificity, %	56.64 (47.79 to 65.49)	52.78 (41.67 to 65.28)	78.76 (71.66 to 85.84)	70.83 (59.72 to 80.56)
PPV, %	53.77 (44.28 to 63.26)	51.43 (39.72 to 63.14)	72.09 (62.61 to 81.57)	63.79 (51.42 to 76.16)
NPV, %	74.42 (65.20 to 83.64)	74.51 (62.55 to 86.47)	83.96 (76.98 to 90.95)	80.95 (71.26 to 90.65)
PLR	1.66 (1.29 to 2.14)	1.56 (1.16 to 2.09)	3.70 (2.54 to 5.37)	2.59 (1.75 to 3.84)
NLR	0.49 (0.34 to 0.71)	0.50 (0.31 to 0.82)	0.27 (0.18 to 0.42)	0.35 (0.21 to 0.57)
Diagnostic accuracy, %	63.02 (55.77 to 69.86)	61.16 (51.87 to 69.88)	78.65 (72.17 to 84.22)	72.73 (63.88 to 80.43)

PPV, Positive predictive value; NPV, Negative predictive value; PLR, Positive likelihood ratio; NLR, Negative likelihood ratio; AUC, area under the receiver operating characteristic curve.

**Figure 4:**
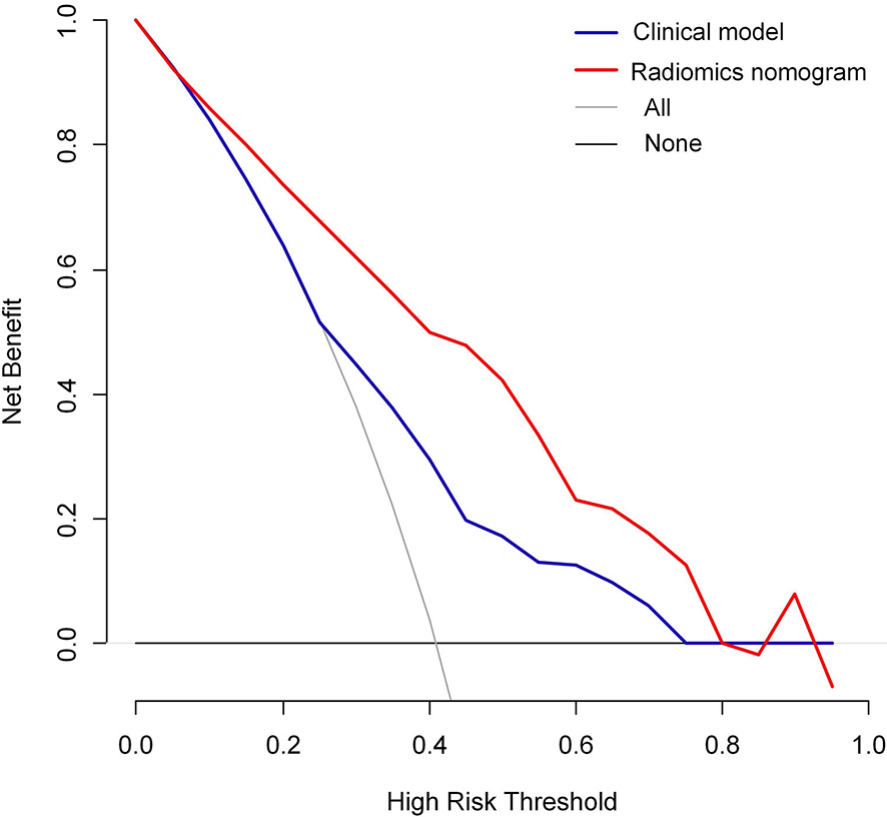
Decision curve analysis (DCA) of the radiomics nomogram and clinical model in predicting the MVI status for hepatocellular carcinoma derived from the all 313 patients.

**Figure 5:**
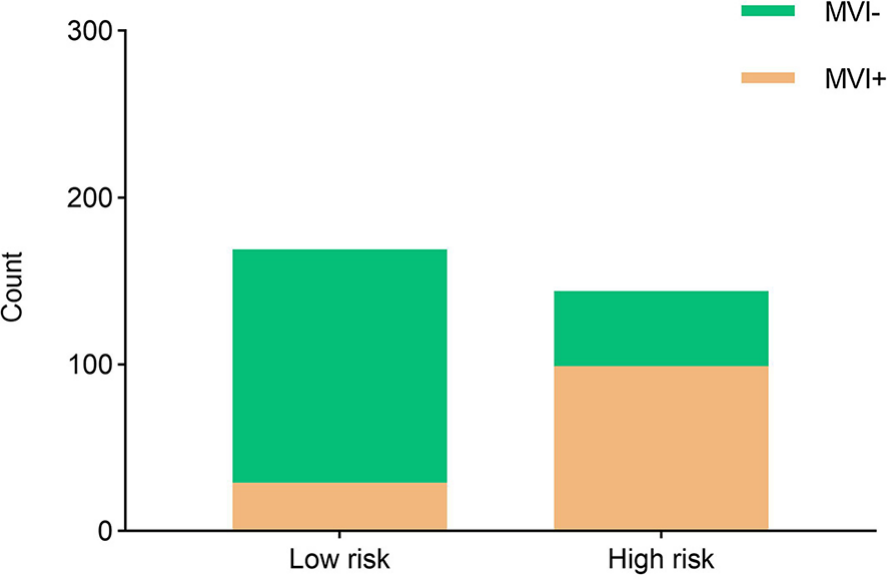
The risk-classification performance of the radiomics nomogram in all 313 patients. MVI+, patients with microvascular invasion; MVI−, patients without microvascular invasion.

Moreover, the radiomics nomogram showed a superior discrimination to the clinical model in the primary dataset (AUC 0.849 *vs*. 0.690, P < 0.001) and validation dataset (AUC 0.788 *vs*. 0.661, P = 0.008) (**Table 3**). The DCA curve demonstrated that using the radiomics nomogram to predict the MVI status was more beneficial than using the clinical model when the threshold probability is between 0.1 and 0.8 (**Figure 4**). In addition, compared with the clinical prediction model which solely incorporated the independent clinical risk predictors, the utilization of the PVP and DP Rad-score significantly improves the prediction performance of the MVI status in terms of the NRI and IDI (**Table 5**). Besides, we further evaluated the performance of the radiomics nomogram in all patients. We classified the 313 patients into high- and low- risk subgroups according to whether the Nomo-score of each patient was above or below the optimal cut-off value (0.452). The results indicated that the high-risk group had a greater proportion of MVI positive in all patients (**Figure 5**). The radiomics nomogram also revealed a more favorable discriminatory ability than the clinical model in all 313 patients (AUC 0.825 *vs*. 0.678, P < 0.001).

**Table 5:** Evaluation of the radiomics nomogram with respect to NRI and IDI.

Characteristic	Primary dataset			Validation dataset		
	Categorical NRI (95% CI)	Continuous NRI (95% CI)	IDI (95% CI)	Categorical NRI (95% CI)	Continuous NRI (95% CI)	IDI (95% CI)
Radiomics nomogram *vs*. clinical model	0.511 (0.344 to 0.678)	0.892 (0.636 to 1.148)	0.240 (0.178 to 0.302)	0.345 (0.132 to 0.557)	0.801 (0.478 to 1.125)	0.185 (0.108 to 0.262)
P-value	<0.0001	<0.0001	<0.0001	0.002	<0.0001	<0.0001

NRI, net reclassification improvement; IDI, index integrated discrimination improvement.

## Discussion

In the current study, we developed and validated a radiomics nomogram that incorporated preoperative CEUS information for the individualized prediction of the MVI status in patients with HCC. The easy-to-use graphic tool might provide useful information to facilitate clinical decision-making. Moreover, our study offers an alternative approach with no radiation while with a comparable performance compared to previous radiomics prediction models based on contrast-enhanced CT (19, 30).

The presence of MVI in patients with HCC is a key determinant associated with adverse tumor biology as well as poor outcomes (5, 31). Furthermore, MVI status has a negative influence on the recurrence and survival rate of HCC patients after transplantation or surgical resection (32, 33). In the Guidelines for Diagnosis and Treatment of Primary Liver Cancer in China (2017 Edition), MVI is an important factor that cannot be ignored in the selection of the treatment plan (34). Partial hepatectomy with a broader resection margin is recommended to improve the recurrence-free survival rate in HCC patients with MVI (21). Therefore, preoperative noninvasive and accurate identification of MVI is very helpful for the preoperative stratification of HCC patients.

In previous studies, some investigators attempted to predict the MVI status of HCC preoperatively by analyzing the clinical risk factors and combined imaging characteristics determined by radiologists (13, 35). However, the difference in the professional knowledge of operators cannot be ignored.

Radiomics utilizes quantitative medical image features to predict tumor biological behavior, providing a new method for the prediction of the MVI status. Hu and his colleagues developed a radiomics strategy based on preoperative grayscale ultrasound images to predict the MVI status of patients with HCC (36). Dong et al. found that gross-tumoral region and peri-tumoral region radiomics signatures based on ultrasound images were also feasible for the preoperative prediction of the MVI status (37). However, their research was only based on grayscale ultrasound images, and did not include image features of other modes. CEUS, which contains imaging information of different aspects of a tumor, is widely used to observe and evaluate microcirculation blood perfusion of liver cancer (23, 38). Therefore, in the present study, we used radiomics strategy to quantitatively extracted multiphase CEUS imaging features to evaluate the overall information related to the MVI status that may be contained in tumors. It is worth noting that the radiomics scores of BM, AP, PVP, and DP were all significantly correlated with the MVI status in the univariate analysis. However, BM and AP radiomics scores were not incorporated in the final radiomics nomogram. We discovered that in the final multivariate logistic regression analysis, the strong discriminatory capacity of the PVP and DP Rad-scores diminished the value of BM and AP Rad-scores. In a previous study, the washout patterns of CEUS in the PVP and DP were considered to be significantly associated with the MVI status. High levels of MVI reduced tumor microvessel density, resulting in a reduced enhancement, that is, the smaller the density of the microvessels, the smaller the amount of contrast agent entering the tumor, which leads to the reduced enhancement on CEUS, promoting washout (26, 39). This might be the reason why our CEUS radiomics signature focused more on the PVP and DP Rad-scores.

As far as we know, our study is the first to utilize the radiomics nomogram to predict the preoperative MVI status of HCC patients based on CEUS imaging. In the current study, tumor size, AFP levels, PVP, and DP radiomics scores were the independent risk predictors associated with the MVI status, and the radiomics nomogram involved the above four factors achieving a favorable predictive value for the MVI status prediction (AUC of 0.849 for the primary dataset and 0.788 for the validation dataset). The predictive calibration curves of the radiomics nomogram in both the primary and validation datasets showed an agreement with the ideal curve. In addition, the significant improvement of NRI and IDI demonstrated that the PVP and DP radiomics signatures may be very useful biomarkers for MVI prediction. Decision curve analysis also proved that the radiomics nomogram can improve the prediction of the MVI status preoperatively. Our CEUS-based radiomics nomogram showed a better discrimination performance compared with nomograms that combined clinical risk factors and imaging features in previous studies (26, 40, 41). Moreover, it is worth noting that in previous studies, all imaging features were based on visual analysis and relied on the subjective evaluation of individual radiologists, while radiomics reflects the texture information of tumor and provides a quantitative analysis of the image features. The nomogram based on the radiomics score is more conducive to the objective evaluation of clinicians of the MVI status.

Our study revealed that a tumor size greater than 5 cm and a preoperative plasma AFP level above 400 μg/L were significant predictive factors associated with the MVI status. Some evidence has suggested that AFP plays an important role in regulating tumor growth and cell differentiation, and may stimulate the proliferation of hepatoma cells through the AFP receptors (42). HCC clones from the same parental cell line showed higher serum AFP levels in nude mice carrying tumor implants with a high metastatic potential than nude mice with low metastatic tumor implants (43). Some previous studies have reported that the preoperative AFP level in HCC patients with MVI were significantly higher, plasma AFP level can be used as an independent predictor to establish a preoperative MVI prediction model (13, 44). A previous study showed that when the diameter of HCC increased, the number of DNA ploidy transformed from diploid to aneuploid increased significantly, and the probability of invasion and metastasis increased (45). The pathological study of Adachi et al. revealed that through the histological examination of surgically resected specimens, portal vein invasion of hepatoma cells was significantly related to tumor size (46). Some studies have also reported that the incidence of MVI increased with an increasing tumor size in HCC (36, 47). The results of our study were consistent with those findings. In the present study, we also constructed a clinical model involving the preoperative AFP level and tumor size. The addition of PVP and DP radiomics signatures to the clinical predict model significantly improved the AUC of the clinical model (from 0.690 to 0.849, 0.661 to 0.788 in the primary dataset and validation dataset, respectively). Moreover, the DCA curve demonstrated that the radiomics nomogram improves the benefit more than the clinical predict model, which implied that radiomics signature added accessorial value to the clinical risk factors in the clinical application. For the clinical application of the radiomics nomogram, we analyzed the sensitivity, specificity, positive and negative likelihood ratios as well as predictive values in evaluating the risk of MVI positive. We displayed that the patients with a total Nomo-score of 0.452 or above were the subgroup of high-risk MVI. Therefore, this subgroup of HCC patients may be more suitable for a larger resection margin during liver resection.

The present study has several limitations. First, the radiomics signature was based on multi-phase CEUS images, and some information might still have been missed in comparison with the CEUS video. It is necessary to further research the association of the radiomics features and video-based CEUS signatures (such as time intensity curve parameters), which may improve the prediction performance of radiomics. Second, this was a retrospective study, so some selection bias and data imbalance may inevitably exist and have influenced our results. In addition, since our research took place in a single institution using one vendor machine, prospective and longitudinal cohort validation with a larger group of patients and multi-vendor machines are still needed to verify the reliability of the developed radiomics nomogram. Third, although all the US examinations were performed by experienced radiologists, there may be heterogeneity in the image quality due to the differences in radiologist manipulation.

## Conclusion

In conclusion, our study developed a non-invasive predictive nomogram that incorporates the radiomics signature of multi-phase CEUS imaging and clinical risk factors, it may provide useful information for the preoperative assessment of the MVI status in patients with HCC and guide a more appropriate surgical planning.

## Data Availability Statement

The original contributions presented in the study are included in the article/**Supplementary Material**. Further inquiries can be directed to the corresponding authors.

## Ethics Statement

The studies involving human participants were reviewed and approved by Ethics Committee of Huazhong University of Science and Technology Drug Clinical Trials. Written informed consent for participation was not required for this study in accordance with the national legislation and the institutional requirements.

## Author Contributions

DZ: Data curation, investigation, methodology, and writing the original draft. QW: Conceptualization, data curation, formal analysis, methodology, and validation. G-GW: Conceptualization and data curation. X-YZ: Formal analysis and visualization. W-WL: Data curation and formal analysis. W-ZL: Methodology, software, validation, and visualization. J-TL: Data curation, project administration, supervision, and writing – review. X-WC: Methodology, resources, supervision, and writing – review. X-JN: Project administration, resources, supervision, and writing – review. CD: Supervision and writing – review. All authors contributed to the article and approved the submitted version.

## Funding

The authors are grateful of the support of National Natural Science Foundation of China (No. 82071953).

## Conflict of Interest

The authors declare that the research was conducted in the absence of any commercial or financial relationships that could be construed as a conflict of interest.

## Publisher’s Note

All claims expressed in this article are solely those of the authors and do not necessarily represent those of their affiliated organizations, or those of the publisher, the editors and the reviewers. Any product that may be evaluated in this article, or claim that may be made by its manufacturer, is not guaranteed or endorsed by the publisher.
