# Supplementary material for: Preoperative Prediction of Microvascular Invasion in Patients With Hepatocellular Carcinoma Based on Radiomics Nomogram Using Contrast-Enhanced Ultrasound
Source: Front Oncol. 2021 Sep 7;11:709339. doi: 10.3389/fonc.2021.709339 (PMC8453164; doi:10.3389/fonc.2021.709339)
Supplement: Supplementary file 1 [file DataSheet_1.docx]

Supplementary Table 1. Major packages of R software used in this study.

| **Functions** | **R package** |
| --- | --- |
| Univariate logistic regression analysis | glm |
| minimum redundancy maximum relevance (mRMR) algorithm | mRMRe |
| least absolute shrinkage and selection operator (LASSO) regression | glmnet |
| draw the receiver operating curve (ROC) and measure the area under the ROC (AUC). | pROC |
| calibration curves | rms |
| Decision curve analysis (DCA) | rmda |

Supplementary Table 2. Performance of the radiomics score for evaluating MVI status.

| Dataset | signature | Cut-off value | AUC (95% CI) | sensitivity (95% CI) | Specificity (95% CI) | Accuracy (95% CI) |
| --- | --- | --- | --- | --- | --- | --- |
| Primary | BM Rad-score | -5.236 | 0.738  (0.667, 0.808) | 0.848  (0.770, 0.924) | 0.531  (0.442, 0.619) | 0.661 (0.590, 0.728) |
| Validation | BM Rad-score |  | 0.710  (0.617, 0.802) | 0.795  (0.673, 0.898) | 0.444  (0.319, 0.556) | 0.587  (0.494, 0.676) |
| Primary | AP Rad-score | -3.404 | 0.750  (0.680, 0.819) | 0.709  (0.608, 0.810) | 0.717  (0.628, 0.796) | 0.714  (0.644, 0.776) |
| Validation | AP Rad-score |  | 0.707  (0.613, 0.801) | 0.633  (0.510, 0.776) | 0.653  (0.542, 0.764) | 0.645  (0.552, 0.730) |
| Primary | PVP Rad-score | -3.191 | 0.801  (0.739, 0.862) | 0.709  (0.608, 0.810) | 0.752  (0.673, 0.823) | 0.734  (0.666, 0.795) |
| Validation | PVP Rad-score |  | 0.751  (0.661, 0.841) | 0.796  (0.694, 0.898) | 0.611  (0.500, 0.722) | 0.686  (0.595, 0.767) |
| Primary | DP Rad-score | -3.525 | 0.750  (0.680, 0.819) | 0.772  (0.684, 0.848) | 0.637  (0.549, 0.717) | 0.693  (0.622, 0.757) |
| Validation | DP Rad-score |  | 0.680  (0.582, 0.777) | 0.714  (0.592, 0.837) | 0.556  (0.444, 0.667) | 0.620  (0.527, 0.707) |


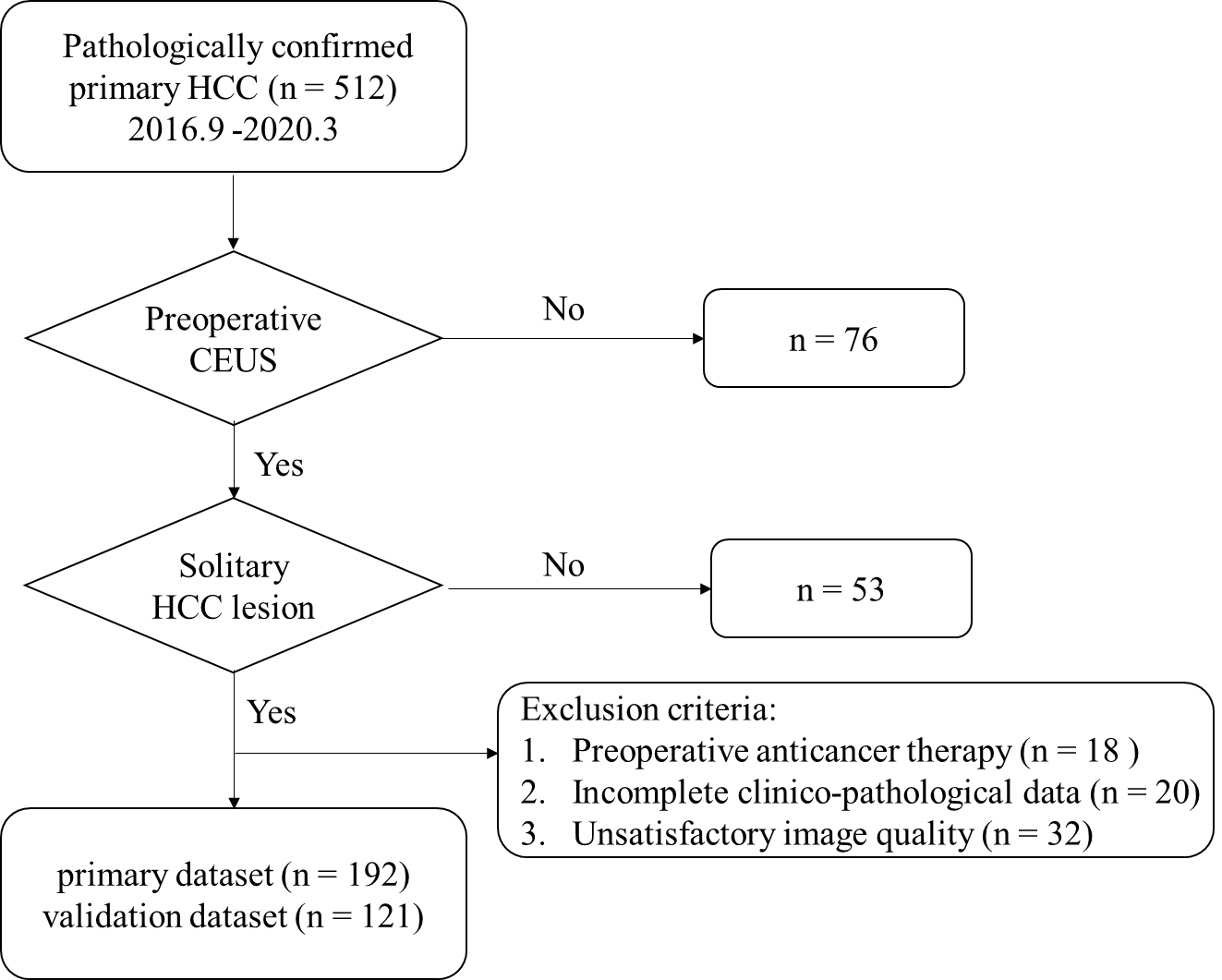


Supplementary Figure A1. Flow diagram of the study population.

**A**

**B**

**D**

**C**

**F**

**E**

**G**

**H**

Supplementary Figure A2. MVI status-related feature selection using the least absolute shrinkage and selection operator (LASSO) logistic regression model in the primary dataset. (A, C, E and G) The five-fold cross-validation and the minimal criteria process was used to generate the optimal penalization coefficient lambda (λ) for the BM (A), AP (C), PVP (E) and DP (G) features in the LASSO model, respectively. LASSO coefficient profiles of the BM (B), AP (D), PVP (F) and DP (H) features.

Supplementary A1. The BM, AP, PVP and DP radiomics score formula.

BM Rad-score = -0.365 + 0.265*original_glszm_SmallAreaHighGrayLevelEmphasis - 0.121*wavelet.LL_ngtdm_Coarseness + 0.006*original_firstorder_Kurtosis + 0.081*wavelet.LH_glrlm_LongRunHighGrayLevelEmphasis + 0.106*wavelet.LH_glszm_LargeAreaHighGrayLevelEmphasis - 0.086*wavelet.LL_glszm_SmallAreaLowGrayLevelEmphasis

AP Rad-score = - 0.355 - 0.104*wavelet.HH_glszm_ZonePercentage + 0.066*wavelet.LL_firstorder_TotalEnergy

PVP Rad-score = -0.365 + 0.306*original_glszm_ZoneEntropy - 0.309*original_shape_Elongation + 0.172*wavelet.HH_gldm_DependenceNonUniformityNormalized -0.236*wavelet.LH_glrlm_LongRunLowGrayLevelEmphasis + 0.149*wavelet.HL_ngtdm_Busyness + 0.196*wavelet.HL_glszm_LargeAreaHighGrayLevelEmphasis -0.107*original_ngtdm_Strength -0.041*wavelet.LH_gldm_SmallDependenceEmphasis

DP Rad-score = -0.358 + 0.291*original_glcm_Idmn -0.175*wavelet.LH_glrlm_LongRunLowGrayLevelEmphasis -0.194*wavelet.HL_glszm_GrayLevelNonUniformityNormalized + 0.223*wavelet.HL_glszm_LargeAreaLowGrayLevelEmphasis -0.105*original_glszm_SizeZoneNonUniformityNormalized -0.070*wavelet.LL_firstorder_Minimum + 0.140*original_ngtdm_Complexity -0.118*wavelet.LL_gldm_SmallDependenceLowGrayLevelEmphasis + 0.055*wavelet.LL_glszm_SizeZoneNonUniformity
